# Lineage Relationships Between Subpallial Progenitors and Glial Cells in the Piriform Cortex

**DOI:** 10.3389/fnins.2022.825969

**Published:** 2022-03-21

**Authors:** Rebeca Sánchez-González, Laura López-Mascaraque

**Affiliations:** Instituto Cajal-CSIC, Madrid, Spain

**Keywords:** glial cells, astrocytes, NG2-glia, clonal analysis, lineage, heterogeneity, olfactory system

## Abstract

The piriform cortex is a paleocortical area, located in the ventrolateral surface of the rodent forebrain, receiving direct input from the olfactory bulb. The three layers of the PC are defined by the diversity of glial and neuronal cells, marker expression, connections, and functions. However, the glial layering, ontogeny, and sibling cell relationship along the PC is an unresolved question in the field. Here, using multi-color genetic lineage tracing approaches with different StarTrack strategies, we performed a rigorous analysis of the derived cell progenies from progenitors located at the subpallium ventricular surface. First, we specifically targeted E12-progenitors with UbC-StarTrack to analyze their adult derived-cell progeny and their location within the piriform cortex layers. The vast majority of the cell progeny derived from targeted progenitors were identified as neurons, but also astrocytes and NG2 cells. Further, to specifically target single Gsx-2 subpallial progenitors and their derived cell-progeny in the piriform cortex, we used the UbC-(Gsx-2-hyPB)-StarTrack to perform an accurate analysis of their clonal relationships. Our results quantitatively delineate the adult clonal cell pattern from single subpallial E12-progenitors, focusing on glial cells. In summary, there is a temporal pattern in the assembly of the glial cell diversity in the piriform cortex, which also reveals spatio-temporal progenitor heterogeneity.

## Introduction

The piriform cortex (PC) is a three-layered paleocortex that receives direct input from the olfactory bulb (OB). From the surface, the PC is formed by a plexiform layer I (LI), divided into a superficial fibrillar layer (LIa) made up of axonal projections of mitral and tufted cells of the OB (the lateral olfactory tract, LOT), and layer Ib (LIb) formed by axonal projections from intracortical pyramidal neurons along with horizontal interneurons (Price, 1973; Neville and Haberly, 2004). Layer II (LII) contains semilunar cells located in the upper part, LIIa (Valverde, 1965; Haberly and Price, 1978), while pyramidal cells are located deeper in LIIb (Haberly and Price, 1978). LIII contains deep pyramidal cells and GABAergic interneurons (Ekstrand et al., 2001; Bekkers and Suzuki, 2013). Below these layers, the ventral part of the claustrum forms the endopiriform nucleus, also known as the deep internal polymorphic layer (Calleja, 1893; Cajal, 1904). Even if PC can be divided into more subregions (anterior and posterior), we will be referring to it as a single structure since previous reports found comparable cell types in both aPC and pPC distributed similarly throughout all PC layers (Martin-Lopez et al., 2019). In addition, we analyzed those sections in which the LOT was evident as a delineated structure, which corresponds to the anterior PC.

Piriform cortex neurons originate at the earliest stages of mouse telencephalic development as revealed through tritiated thymidine (Hinds and Angevine, 1965; Bayer, 1986; Valverde and Santacana, 1994) and BrdU experiments (De Carlos et al., 1996; Nomura et al., 2006) in an inside-out sequence (Luzzati, 2015; Klingler, 2017). Early in development (E10–E11), different pallial areas give rise to cells that migrate tangentially along diverse routes, to gather into the olfactory cortex (García-Moreno et al., 2008; Ceci et al., 2012). In the PC, most pallial progenitors differentiate into inhibitory neurons, while those from subpallium, into excitatory neurons (García-Moreno et al., 2008). Later in development, both pallial and subpallial ventricular zones produce cells that migrate radially toward the PC (Carney et al., 2006; Shapiro et al., 2007; García-Moreno et al., 2008), although most neurons and astrocytes in the PC originate from ventral progenitor cells (Tsoa et al., 2014; Torigoe et al., 2015). In addition, the multicolor lineage tracing approach based on the expression of three reporter proteins showed a committed cell potency of E11-progenitors, located at the lateral ganglionic eminence (LGE), to give rise to cells clustered in a specific laminar location (Martín-López et al., 2013). Dbx1-expressing neural progenitors preferentially contribute to LII, being enriched in the ventral PC (Shabangu et al., 2021). Transgenic mice models showed that NG2+ /PDGFRα+ proteolipid protein promoter-expressing progenitors generate pyramidal glutamatergic neurons in adult PC (Rivers et al., 2008; Guo et al., 2010). However, other studies reported that NG2-positive cells do not produce new neurons using genetically modified mice [Dimou et al. (2008), Komitova et al. (2009), reviewed by Nishiyama et al. (2009) and Richardson et al. (2011)].

So far, while the developmental aspects of neurons in the PC are well characterized, the development and clonal analyses of glial cells types is less understood. Our study is focused on the cell progeny derived from E12-progenitors corresponding to the onset of the glial production in subpallial areas (Kessaris et al., 2006; Martín-López et al., 2013; Torigoe et al., 2015). Here, in order to label the complete progeny in the adult PC derived from individual subpallial E12-progenitors, we employed two separate StarTrack strategies with either the hyperactive transposase, the UbC-StarTrack (Figueres-Oñate et al., 2016), or the Gsx-2 transposase, the UbC-(Gsx-2-PB)-StarTrack (Sánchez-González et al., 2020a) of the PiggyBac system. These approaches highlight the laminar distribution of the glial cells in the PC and their clonal relationships. The StarTrack strategy, driven by a Gsx-2 promoter, ensures the specific targeting of subpallial progenitors located within the LGE (Corbin et al., 2000; Yun et al., 2001). Then, we analyze the cell fate of barcoded subpallial progenitors to determine the cell dispersion and identity of sibling cells and their clonal relationships along with the PC, focusing on glial cells. Our results revealed neural cells along the PC with different cellular identities, such as neurons, astrocytes, oligodendrocytes, and NG2 cells.

## Materials and Methods

### Animals

Pregnant C57/BL6 mice, obtained from the animal facility of the Cajal Institute, were kept under standard housing conditions. All animal procedures were carried out following the European Union guidelines on the use and welfare of experimental animals (2010/63/EU) and those of the Spanish Ministry of Agriculture (RD 1201/2005 and L 32/2007). All the experiments were approved by the CSIC Bioethical Committee (PROEX223/16). The day of vaginal plug was considered as the first embryonic day (E0) and the day of birth was considered as postnatal day 0 (P0), and in all the experiments, a minimum of *n* = 3 animals were considered for each condition.

### StarTrack Plasmids

We used UbC-StarTrack constructs (Figueres-Oñate et al., 2016) along with either the hyperactive transposase (CMV-hyPBase) or the Gsx-2 transposase (Gsx-2-hyPBase) of the PiggyBac system (Sánchez-González et al., 2020a). All the plasmids were sequenced to confirm successful cloning (Sigma–Aldrich; Merck KGaA, Darmstadt, Germany). To avoid the episomal copies Cre-ERT2, plasmid was injected along with UbC-StarTrack mixture. Moreover, the 12 UbC-StarTrack vectors were injected along with Gsx-2-hyPBase and Cre-ERT2 to target the E12 subpallial progenitor cells with the active Gsx-2 promoter in an independent approach.

### *In utero* Electroporation

*In utero* electroporation was performed in pregnant mice at E12 as described previously (Figueres-Oñate et al., 2016; Sánchez-González et al., 2020a). Briefly, a mixture of plasmids (1–2 μg/μl) and 0.1% Fast Green was injected into the LVs of E12 embryos using a glass micropipette. The injection was guided using an ultrasound device (VeVo-770; VisualSonics, Toronto, ON, Canada). After plasmid injection, all embryos were electroporated with electrode paddles using five pulses of 33 V during 50 ms every 950 ms. The positive electrode was positioned on top of the ventro-rostral areas of the embryonic telencephalon to direct the negatively charged DNA to the subpallial zone. After the five pulses, the electroporated animals were placed again into the abdominal cavity of the pregnant mice and reanimated for several minutes on a 37°C heating plate. The pregnant mice were monitored for 3 days in a row and administrated meloxicam and Baytril during the surgery and the next 2 days after the procedure. A single dose of Tamoxifen (Tx, Merck KGaA, Darmstadt, Germany, 5 mg/40 g body weight) was intraperitoneally administered in P3–P5 pups to eliminate episomal copies of the UbC-StarTrack plasmids (Figueres-Oñate and López-Mascaraque, 2016). The electroporated mice were analyzed from P30 onward to analyze the derived cell progeny at young adult ages (at least three animals per experimental group).

### Immunohistochemistry

Animals, anesthetized with pentobarbital (Dolethal, 40–50 mg/kg), were perfused with 4% paraformaldehyde (PFA) in 0.1 M phosphate buffer (PB). Afterward, the brain was removed and placed overnight in small tubes with 4% PFA in 0.1 M phosphate buffer (PB). Serial vibratome brain sections (50 μm thick) were stained for different neural markers as follows. Slices were permeabilized with PBS containing Triton X-100 (PBS-T) and then incubated in blocking solution [5% normal goat serum (NGS) in PBS-T 0.1%]. Sections were then incubated with rabbit polyclonal anti-Olig2 (Millipore-AB9610), rabbit polyclonal anti-PDGFRα (Cell Signaling-3169), rabbit polyclonal anti-GFAP (Dako-31745), mouse monoclonal anti-S100β (Abcam-Ab66028), anti-Adenomatous Polyposis Coli (APC; Calbiochem-OP80, Doublecortin (DCX; Cell Signaling-4604), and anti-Neuronal Nuclei (NeuN; Millipore- MAB377) O/N at 4°C. After at least three washes with PBST 0.1%, the sections were incubated for up to 2 h with Alexa far-red goat anti-rabbit or goat anti-mouse IgG (1:1.000, Alexa Fluor 633 or 647, Molecular Probes). Finally, the sections were washed several times with PB, mounted from the most rostral to caudal slides onto glass slides with Mowiol, coverslipped, and observed in an epifluorescence microscope (Eclipse E600; Nikon, United States).

All sections were examined under the epifluorescence microscope equipped with the appropriate filter cubes (Semrock, IDEX Health & Science, United States): UV-2A (FF01-334/40-25), GFP (FF01-473/10), mCherry (FF01-590/20), and Cy5 (FF02-628/40-25). Afterward, images were finally acquired on a TCS-SP5 confocal microscope (Leica Microsystems, Wetzlar, Germany) using a 20× magnitude, with the wavelength confirmation as described previously. Confocal laser lines were maximal around 40% in all samples. Maximum projection images were analyzed using LASAF (Leica Software). All stitching and contrast adjustments were performed with LASX software and Fiji software ImageJ. Selected brain sections were stained with DAPI for 15 min before mounting to delineate the PC.

### Quantification and Clonal Analysis

For each experiment, cells were counted in serial sections with the ImageJ Cell Counter Plugin along the *Z*-axis. Afterward, the proportion of those cells in the anterior PC was calculated per each neural cell type and location (LOT, PC, and other ventral regions). For statistics, GraphPad Prism 6.0 (GraphPad, San Diego, CA, United States) was used, and the statistical significance between two groups was assessed with two-tailed unpaired Student’s *t*-tests and one-way analysis of variance (ANOVA) for multiple comparisons between the groups. The values were represented as mean ± SEM along with the experimental data. A confidence interval of 95% (*p* < 0.05) was determined for the statistically significant values. Critical values of *p < 0.05, ^**^p < 0.01, and ^***^p < 0.001 were adopted to determine statistical differences. Graphs were obtained using Excel Office, GraphPad Prism 6.0 (San Diego, CA, United States), and CorelDRAW Graphic Suite 2018 (Corel Corporation, Ottawa, ON, Canada).

Clonal analysis was performed using a macro for Image J in serial sections disposed from the most rostral areas to the caudal sections with the purpose to organize clones in the rostro-caudal axis (Figueres-Oñate et al., 2016; Sánchez-González et al., 2020b). In brief, a barcode was created such as a binary signature (0 = absence, 1 = presence of cytoplasmic and nuclear marker of YFP, mKO, mCerulean, mCherry, mTSapphire, and EGFP) in all the animals analyzed. Finally, the cells sharing the same combination/location of fluorophores/signature were cataloged as clones after classifying all the labeled cells in the rostro-caudal axis. The specific criteria to catalog a clone were performed according to previous approaches (Figueres-Oñate et al., 2019; Sánchez-González et al., 2020b). The final dispersion of the sibling cells was calculated taking into account the distance from those cells in the rostro-caudal axis containing cells of that clone.

## Results

### Specific Targeting of Subpallial Progenitor Cells Using New StarTrack Approaches

**FIGURE 1:**
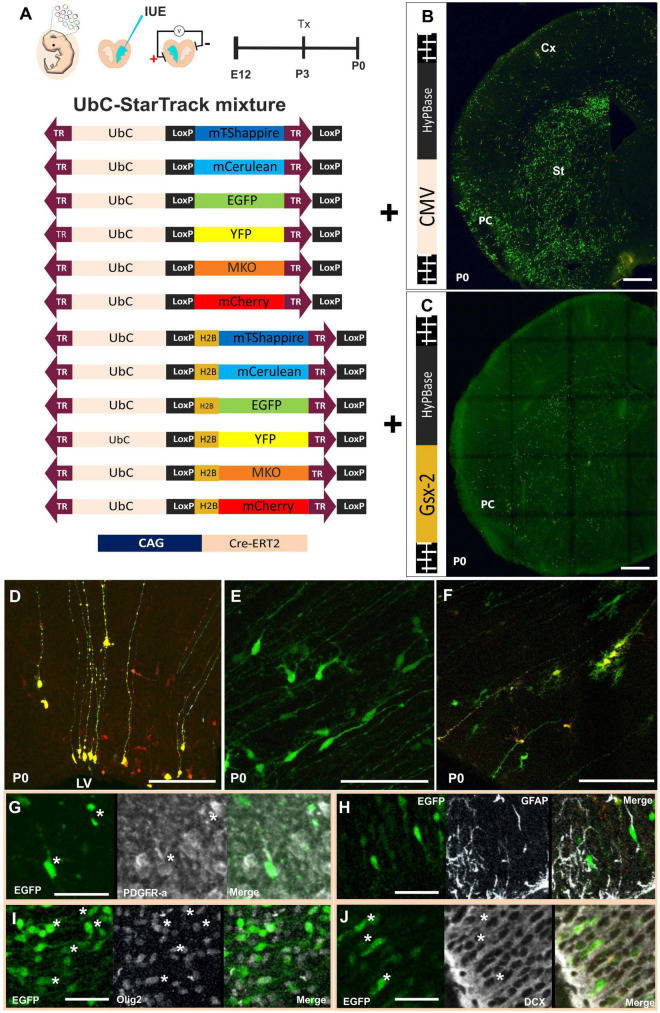
Specific targeting of subpallial progenitor cells using new StarTrack approaches. **(A)** Scheme of ventral IUE: injection of UbC-StarTrack mixture using **(B)** CMV-transposase, showing an image of the labeled cells at P0, or **(C)** Gsx-2 transposase and their derived-labeled cell progeny at P0. **(D–F)** Cell progeny derived from E12-progenitors located close to the ventricular zone with similar morphologies to radial glial cells **(D)**, immature neurons and glial progenitor cells **(E,F)** at P0 using either UbC-StarTrack or UbC-(Gsx-2-PB)-StarTrack. These cells co-expressed different markers such as PDGFR-α **(G)**, GFAP **(H)**, Olig2 **(I)**, and DCX **(J)** showed with asterisks (*). IUE, *in utero* electroporation; LV, lateral ventricle; PC, piriform cortex; St, striatum; Cx, cerebral cortex; Tx, tamoxifen. Scale bar: 100 μm **(B,C)**; 50 μm **(D–J)**.

In an attempt to reveal the relationships underlying the familiar arrangements of neural cells in the PC, we used the 12 UbC-StarTrack plasmids at E12 mouse embryos along with Cre-ERT2 recombinase that allows us to remove the episomal copies due to the action of the tamoxifen at P3 (Figure 1A). Firstly, the UbC-StarTrack mix with the CMV-hyperactive Piggybac transposase was used to target the ventral progenitor cells and their cell-derived progeny (Figure 1B). At early stages, such as P0, the labeled cells were located in the striatum (St), ventral cortex, and even dorsal cortex (Figure 1B). With the purpose to test the cell-derived progeny of specific subpallial progenitors, the UbC-StarTrack strategy was modified by combining the 12 UbC-StarTrack constructs with a new transposase under the control of a Gsx-2 promoter, named as UbC-(Gsx-2-PB)-StarTrack (Figure 1C), then the Gsx-2 promoter allows transposase-mediated genomic integration of these constructs into subpallial Gsx-2-active progenitors. Using this strategy, the cell-derived progeny was also located in the striatum and ventral cortex (Figure 1C), but not in the dorsal (pallial) cortex, as occurs with the UbC-StarTrack (Figure 1B).

The immature morphologies of ventricular labeled cells were similar, independent of the transposase (Figures 1D–F). The progenitor cells targeted with UbC-StarTrack can be identified as radial glial cells (RGCs) with long ascending processes (Figure 1D), and also immature morphologies of neuroblasts and glial cells (Figures 1E,F). Progenitor cells coexpressed different markers such as PDGFR-α (Figure 1G), GFAP in some of the labeled cells with similar morphologies to RGCs (Figure 1H), and even Olig2 in other labeled cells (Figure 1I). Finally, labeled neuroblasts in layer II were identified by the co-expression of DCX (Figure 1J). So, the different strategies of the UbC-StarTrack method allow us to precisely determine the fate potential of specific and single progenitor cells at the embryonic, perinatal, and postnatal stages.

### Cell Fate of Ventral Progenitors in Piriform Cortex

**FIGURE 2:**
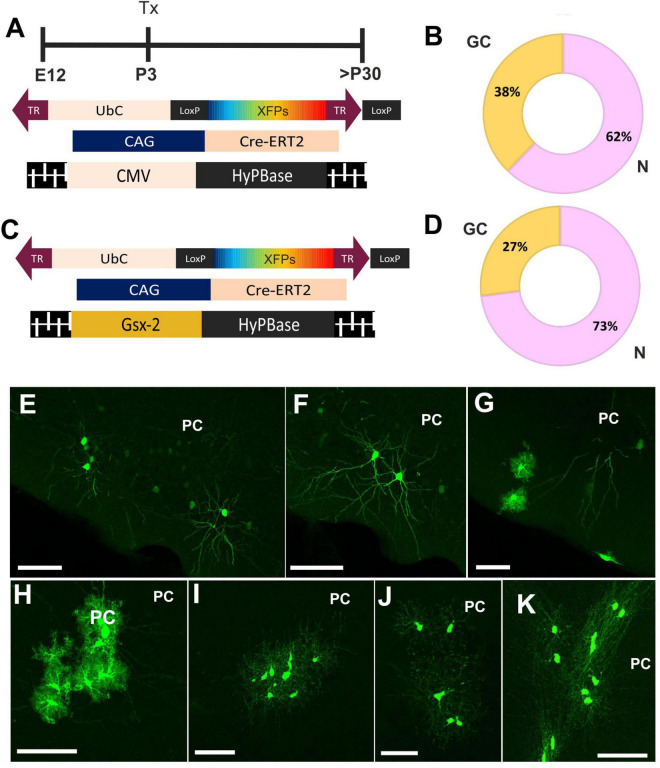
Cell fate of ventral progenitors in adult piriform cortex. **(A)** Scheme of the UbC-StarTrack approach using the CMV transposase. **(B)** The proportion of neurons (pink) was 62% whereas in glial cells (yellow) the proportion was 38%. **(C)** Gsx2-trasnposase was used to target ventral E12 progenitor cells and their cell progeny was analyzed in adult stages. **(D)** The proportion of their derived-cell progeny was similar to panel **(B)**. **(E,F)** Labeled neurons are located in LII-LIII, as well as the glial cell profiles **(G)**. **(H)** Astrocytes with stellate morphology located along the different PC layers. **(I,J)** NG2 cells in the PC. **(K)** OL cells labeled in the PC. N, neurons; GC, glial cells, OL, oligodendrocytes; IUE, *in utero* electroporation; LOT, lateral olfactory track; PC, piriform cortex; Tx, tamoxifen. Scale bar: 50 μm.

To address the fate potential of E12-progenitor cells, in young adult PC, we performed two strategies of UbC-StarTrack (Figure 2). First, we targeted ventral E12-progenitors using UbC-StarTrack to identify the different neural cells located in PC at early adult stages (Figure 2A). We performed a quantitative analysis of the progeny, using a total of 6,680 cellular profiles, located along the different layers of the PC. The identification of cell types was performed first by morphological criteria and then with immunohistochemistry against specific markers. About 62% of these cells were classified as neurons in LII–LIII (Figures 2B,E,F), while the 38% comprised glial cells spread across LI–LIII (Figures 2B,G), including the LOT. A total of 18% was identified as astroglial profiles (Figures 2G,H), while the 9%, as NG2 cells (Figures 2I,J). Just 2% of cells were identified as oligodendrocytes (Figure 2K).

Next, we performed a comparative analysis of the cell progeny derived from ventral E12-progenitors using UbC-(Gsx-2-PB)-StarTrack (Figure 2C) to compare with the above results using the UbC-StarTrack (Figures 2A,B). A total of 4,585 labeled cells were counted, where 73% were identified as neurons and 27% corresponded to glial cells (Figure 2D). The proportion of glial cell-derived progeny of Gsx-2-progenitor cells was similar using both approaches, the proportion of astrocytes (15%) being higher than NG2 cells (7%). Just 1% of labeled cells were identified as oligodendrocytes (data not shown). Then, the cell progeny derived from ventral progenitor cells at E12 comprises different neural cell types equivalent with morphological features characteristic of neurons (Figures 2E,F), neural cells in the LOT, astrocytes (Figure 2H), NG2-glia (Figures 2I,J), and oligodendrocytes (Figure 2K) across the PC layers.

**FIGURE 3:**
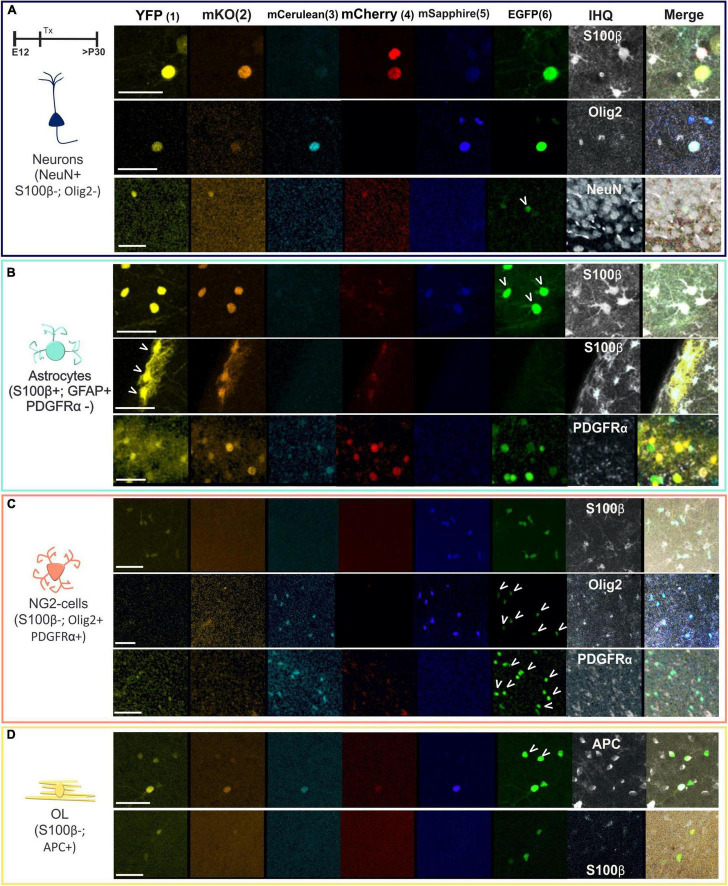
Immuno-characterization of neural cells located in the piriform cortex. **(A)** Cells expressing NeuN but without S100β and Olig2 expression were identified as neurons. **(B)** Positive cells to S100β and GFAP but not to PDGFRα were classified as astrocytes. **(C)** NG2 cells expressed PDGFRα and Olig2 but not S100β. **(D)** Oligodendrocytes were identified with APC. Arrowheads pointed the co-expression of UbC-(Gsx-2-PB)-StarTrack with the antibodies. The six reporter proteins of UbC-(Gsx-2-PB)-StarTrack acquired in separated channels of the confocal are the monomeric Turbo Sapphire fluorescent protein (mT-Sapphire: blue), monomeric Cerulean fluorescent protein (mCerulean: soft blue), enhanced green fluorescent protein (EGFP: green), yellow fluorescent protein (YFP: yellow), monomeric Kusabira Orange fluorescent protein (mKO: orange) and monomeric Cherry fluorescent protein (mCherry: red). The far red was used for the immunohistochemistry and is represented in gray. Schematic neurons are in blue; astrocytes in green; NG2 cells in soft red and OL in yellow. OL, oligodendrocytes. Scale bar: 25 μm.

Even with the clear morphological differences and criteria used to recognize neurons, astrocytes, oligodendrocytes, or NG2 cells, we used immunohistochemical specific markers to confirm their cell identity. Since a specific marker varies significantly in its sensitivity or specificity and may not label the entire population of a given cell type, we used different markers to discard or to assign the cell identity. Each immunomarker was used in one section since we only have the far red channel to perform the immunostainings because the rest of the fluorescent channels are occupied by the different reporter proteins of the StarTrack mix. Then, we used serial sections to separately perform the immunostaining of the different antibodies. These labeled cells were positive to NeuN, negative to Olig2 or S100β (Figure 3A), and classified as neurons. Other cells co-labeled with S100β or GFAP, but not PDGFRα, were identified as astrocytes (Figure 3B). The labeled cells that expressed Olig2 or PDGFR-α but were negative to S100β were identified as NG2 cells (Figure 3C). Finally, oligodendrocytes were characterized as APC positive cells but negative to S100β (Figure 3D).

Our data allowed us to validate the subpallial targeting of progenitors using both StarTrack approaches, concluding that after performing separate UbC-StarTrack approaches, the cell-derived progeny of these progenitors spread into the three layers of PC and showed different neural phenotypes.

### Clonal Analysis of the Progeny Derived From Single Subpallial Progenitors

**FIGURE 4:**
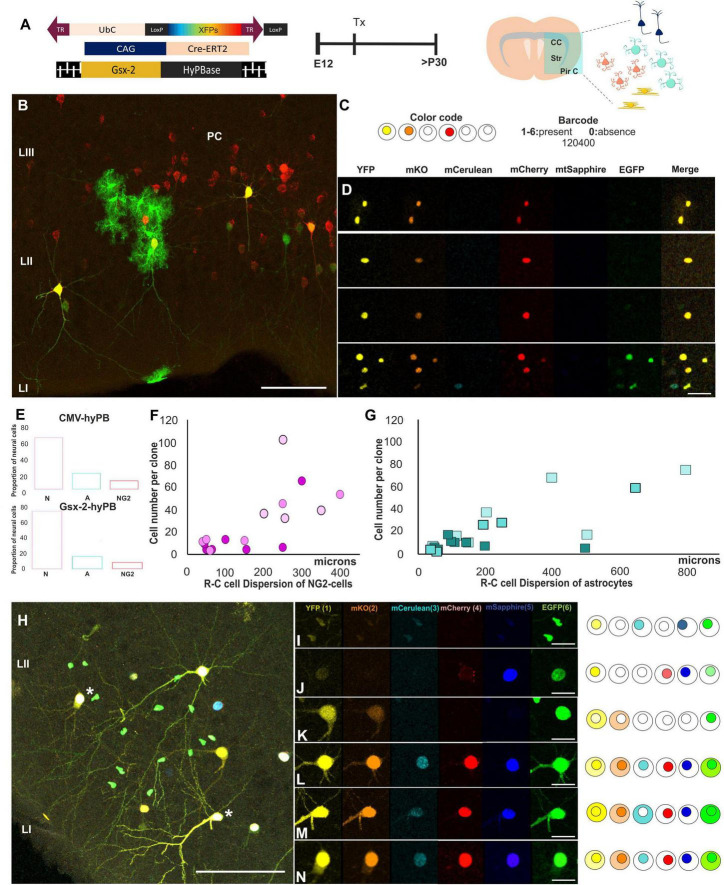
Clonal analysis of the progeny derived from single subpallial progenitors. **(A)** Diagram of the UbC-StarTrack approach. **(B–D)** StarTrack labeled cells located in the PC and their clonal timeline. The color code of sibling cells located in the PC were analyzed by the presence (1–6) or absence of the reporter proteins. **(E)** The proportion of glial cells using both UbC-StarTrack approaches show similar proportion of both astrocytes and NG2 cells. **(F,G)** Sibling NG2-glia cells dispersed less than astrocytes in the PC (*y*-axis), but the number of cells per clone was higher (*x*-axis) than astrocytes. **(H)** Neural cells were localized along the PC in different layers and with different morphologies. Different barcodes of these neural cells are detailed from panels **(I–N)**, where neurons L and N share the same reporter protein combination concluding that they derived from the same E12-progenitor cell. NG2 cells are in pink and astrocytes in blue. N, neuron; A, astrocytes; PC, piriform cortex; R-C, rostro-caudal; Tx, tamoxifen. Scale bar: 50 μm **(B,H)** and 25 μm **(D,I–N)**.

Afterward, to assess the clonal relationships of the early adult progeny derived from E12-subpallial progenitors, we performed IUE using UbC-(Gsx-2-PB)-StarTrack (Figure 4A). Sibling neural cells located in the PC (Figure 4B) were analyzed by their cell type, the number of cells per clone (Figure 4E), position across the PC layers (Figure 4B), and dispersion along the rostro-caudal (R-C) axis (Figures 4F,G). Moreover, the barcode for each clone was established based on the color code, location of the reporter protein, and intensity of sibling cells in the PC (Figures 4C,D). The number of glial cells was lower than neurons in the different experiments (Figure 4E). The dispersion of labeled glial cells along the R-C axis was lower in NG2 glia (Figure 4F) than astrocytes (Figure 4G) in young adult brains. Three different animals were used for each approach, represented in different pink/green patterns to confirm a similar dispersion of the samples. Since the proportion of labeled oligodendrocytes in the PC was less than 2%, we excluded these cells in the clonal analysis. Otherwise, clonally related astrocytes dispersed up to 800 microns in the PC (Figure 4G), whereas NG2 cells occupied up to 400 microns (Figure 4F). Although NG2 cells formed bigger clones than astrocytes, their averages were similar due to the variability in the number of cells per clone in both sibling NG2 cells and astrocytes (Figures 4F,G). The larger clone of NG2 cells was formed by 102 cells while the smallest was formed by three cells. However, the bigger astrocyte sibling group was formed by up to 60 cells, and the lower, up to two cells.

Furthermore, the lack of mixed clones formed by astrocytes and NG2 cells suggests the lack of bipotent progenitors at E12. Thus, in the early adult PC, the progeny derived from E12-progenitors showed different barcodes depending on their sibling relationships (Figures 4H–N). In Figure 4H, neurons displayed similarities in terms of their morphology and color profile; nonetheless, the clonal relationships of cells were specifically examined using the color, location in the cytoplasm and nucleus, and intensity of each fluorophore (Figures 4I–M). Concerning sibling neurons, we also observed clonal arrangements of neurons that displayed the same barcode (Figure 4H, asterisks), analyzed channel by channel (Figures 4L,N). However, the surrounding neural cells showed different barcodes, and in consequence, they were not belonging to the same clone, independent of their lineage.

In summary, these results evidenced the existence of committed ventral E12-progenitors that are capable of giving rise to either glial cells or neurons. In addition, sibling astrocytes located in the PC formed clusters with larger R-C dispersion than NG2-sibling cells.

### Layer Distribution of Astrocytes and NG2 Cells in the Piriform Cortex After Targeting E12-Progenitors

**FIGURE 5:**
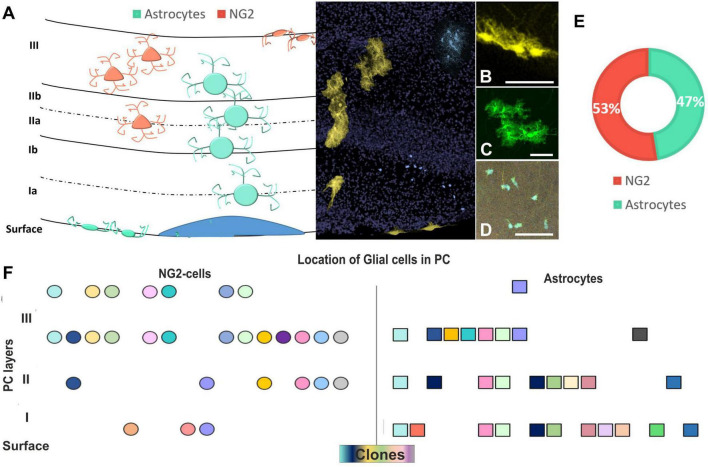
Layer distribution of astrocytes and NG2 cells in the PC after targeting E12-progenitors. **(A)** Graphical scheme of glial cells location across the PC layers. Layer identification of the PC with DAPI (in gray) and UbC-StarTrack labeled cells shows the location of cells in the PC at P30. **(B)** Labeled cells with UbC-StarTrack along PC displayed discrete clusters of cells distributed from the pial surface **(B)** to lower cortical layers **(C,D)**, corresponding to pial astrocytes **(B)**, protoplasmic astrocytes **(C)**, and NG2-glia **(D)**. **(E)** The proportion of NG2 (53%) and astrocyte clones were similar (47%) in the three analyzed mice. **(F)** The location of clonally related cells in the PC shows clones of NG2 cells dispersed preferentially in layers II–III, while astrocyte clones dispersed along the different layers. NG2 cells are in soft red and astrocytes in blue/green. The NG2 clones are represented in circles and astrocytes are represented in squares. The different colors indicate each cluster of sibling cells. PC, piriform cortex; St, striatum; CC, corpus callosum. Scale bar: 50 μm.

After targeting E12-progenitors using UbC-(Gsx-2-PB)-StarTrack, glial cells were located in different layers of the PC (Figure 5) in young adult mice. Those cells were classified attending to their cell identity, morphology, and location within the PC layers (Figure 5A). Labeled astrocytes were either located in LI, named as pial astrocytes (Figures 5A,B), or protoplasmic astrocytes distributed across the PC layers (Figures 5A,C). However, NG2 cells spread throughout the PC (Figures 5A,D), although mostly restricted to LII and LIII, close to the deep internal polymorphic layer (Figures 5A,F). The proportions of astrocytes (47%) and NG2 cells (53%) derived from E12 progenitors were similar (Figure 5E), although the R-C dispersion was larger in astrocytes than in NG2 cells (Figures 4F,G). Hence, even if the glial cells were distributed along the different PC layers, clonally related astrocytes occupied several layers of the PC, whereas sibling NG2 cells are located in t layers II–III of the PC (Figure 5F). Besides, glial cells are also situated around neurons in LII but they were not found any sibling neuron with glial cells.

In summary, E12 progenitors produce clones in PC specifically committed to generating neuron, astrocytes, and NG2 cells, with a low proportion of oligodendrocytes, with precise dispersion patterns.

## Discussion

The present study provides new information about the clonal organization in the PC mainly focused on glial cells derived from E12 subpallial progenitors using two different UbC-StarTrack approaches. These approaches allow unraveling the fate potential of individual progenitors and the clonal relationships of their progeny in the PC. In addition, they permit exploring how crucial the spatio-temporal ontogenic origin is to gain new insights regarding progenitor heterogeneity. While the laminar organization, development, and cellular properties of neurons in the PC had been well characterized (Bayer, 1986; Valverde and Santacana, 1994; Carney et al., 2006; García-Moreno et al., 2008; Sarma et al., 2011; Martin-Lopez et al., 2019; Shepherd et al., 2021), many aspects related to glial cell types remained unknown. Our results unravel the laminar distribution of both NG2 cells and astrocytes, revealing that subpallial E12-progenitor cells are committed to giving rise to different glial cell lineages. Regarding oligodendroglial lineage, our data showed a lower number of mature oligodendrocytes, probably related to the progenitor targeted stage. Targeting later embryonic progenitor stages produces a higher number of cells due to the great majority of myelin-forming oligodendrocytes born at the early postnatal stages (Kessaris et al., 2008; Clarke et al., 2012; Young et al., 2013).

Previous research has shown that LGE progenitor cells are capable of giving rise to astrocytes in adult PC due to the asymmetric division of targeted progenitor cells (Martin-Lopez et al., 2019). Here, we demonstrate the laminar preference of astroglial clones verifying their layer restriction of cell lineages in the PC. We revealed the distribution of astrocytes along the PC layers as well as the NG2 cells that were preferentially located in LIII. Moreover, we showed astroglial clones widely dispersed in the R-C axis, while NG2 cell displayed lower dispersion. However, the number of sibling cells per clone was lower in astroglial clones than in NG2 clones, although not as big as those described in pallial NG2 clones (García-Marqués et al., 2014; Sánchez-González et al., 2020b). In addition, our glial clonal analysis, targeting ventral embryonic progenitors, displayed different cell dispersion patterns and clonal size than previous lineage tracing analysis at postnatal stages (Sánchez-González et al., 2020b). Hence, the number of NG2 cells increased with age, which could be crucial to support new therapies underlying cell aging.

The evidence that astrocytes in the PC can be associated with neurons and astrocytes, derived from a distinct progenitor domain, was reported using Nkx2-Cre-mice and IUE (Torigoe et al., 2015). Furthermore, the new UbC-(Gsx-2-PB)-StarTrack (Sánchez-González et al., 2020a) is accurate in the ventral targeting of progenitor cells, displaying a similar proportion of neural cells located in the PC. The source of PC neural cells, had been reported from multiple origins in the ventricular surface [reviewed by Klingler (2017)]. Otherwise, the migration pathways implicate the tangential and radial process of cell routes. Opposite to what occurred in the dorsal pallium, in the PC, some neurons from the pallium differentiate in inhibitory neurons, and some neurons from the subpallium, in excitatory neurons (De Carlos et al., 1996; García-Moreno et al., 2008). This diversity implies that cells from different areas of the ventricular surface can be correlated with their heterogeneous identities and functions in odor processing. Actually, some approaches aim at the connectivity properties of the cells with their molecular identity (Diodato et al., 2016). Accordingly, the expression of voltage−dependent K+ channel, Kv4.3, is clonally related in cortical astrocytes in a layer−specific manner, with all sibling cells being either positive or negative for Kv4.3 (Götz et al., 2021). Moreover, sibling astrocytes showed preferential gap-junction coupling compared to non-related astrocytes (Gutiérrez et al., 2019). This coupled response between sibling cells may be implicated in physiological and pathological conditions (Martín-López et al., 2013; Bribián et al., 2018; Barriola et al., 2020). Therefore, this data reinforces the idea of the high heterogeneity of these neural cells at a morphological, functional, and genetic level.

Nevertheless, NG2 cells were originally assumed to be a homogeneous type of progenitors of oligodendrocytes, known as oligodendrocyte precursor cells (OPCs). However, these cells are now considered as the fourth glial cell type with astroglia, microglia, and oligodendroglia (Nishiyama et al., 2009; Dimou and Gallo, 2015). This is supported in base to either their electrical properties (Chittajallu et al., 2004; Karadottir et al., 2005), proliferation rates (Psachoulia et al., 2009), response to injury (Keirstead et al., 1998; Lytle et al., 2009), and their abilities to differentiate into myelinating OLs (Mallon et al., 2002; Dimou et al., 2008) or other neural cells, including neurons (Rivers et al., 2008; Guo et al., 2010; Zhu et al., 2011). Previous *in vitro* studies indicated that a small subpopulation of adult NG2 cells in layer II of PC expressed markers of immature neurons (Gómez-Climent et al., 2008; Rubio et al., 2016). Although their capability to generate neurons is not yet fully understood (Richardson et al., 2011), it could be relevant for neurodegenerative diseases and aging, concerning their stem cell-like characteristics or their reprogramming capabilities (Heinrich et al., 2014; Torper et al., 2015; Magnusson and Frisén, 2016; Pereira et al., 2017; Tsunemoto et al., 2018). That capacity could be one of the sources of the endogenous precursors to generate new neurons in the PC where NG2 cells displayed a highly plastic dynamic to differentiate into pyramidal neurons in the mouse (Guo et al., 2010). Indeed, some reports showed after experimental ablation, and the replacement neurons can extend long-range axons toward their original targets (Roig-Puiggros et al., 2020).

Therefore, this heterogeneous disposition of astrocytes and NG2 cells in the PC could imply the multifunctional properties of these cells (Dimou and Gallo., 2015; Götz et al., 2021; Herrero-Navarro et al., 2021). Furthermore, it allows understanding the importance of glial cells in this dynamic cortical area that receives the information from the OB and its role in odor coding related to the cortical sensory processing. The sibling cell relationships and their dispersion in the PC are related to the dynamic formation of the olfactory system along with life, which is important to decipher the PC ontogeny.

To advance our understanding of the paleocortex, it is necessary to improve the methodology that leads to the specification of the cell diversity in the PC. Therefore, additional multi-omics technologies might provide new insights related to dynamics, behavior, heterogeneity, and functionality of PC neural cell types.

## Data Availability Statement

The original contributions presented in the study are included in the article/supplementary material, further inquiries can be directed to the corresponding author.

## Ethics Statement

All the experiments were reviewed and approved by the CSIC Bioethical Committee (PROEX223/16).

## Author Contributions

LL-M conceived, designed, and supervised the research. RS-G performed experiments and analyzed the data. Both authors wrote and approved the final manuscript.

## Conflict of Interest

The authors declare that the research was conducted in the absence of any commercial or financial relationships that could be construed as a potential conflict of interest.

## Publisher’s Note

All claims expressed in this article are solely those of the authors and do not necessarily represent those of their affiliated organizations, or those of the publisher, the editors and the reviewers. Any product that may be evaluated in this article, or claim that may be made by its manufacturer, is not guaranteed or endorsed by the publisher.
